# Resources and challenges for midwives supporting women in the latent phase of labor: A qualitative study from Germany

**DOI:** 10.18332/ejm/221144

**Published:** 2026-05-31

**Authors:** Caro Jeltsch, Sabine Rivière, Oda von Rahden

**Affiliations:** 1 Department of Technology and Health for People, Jade University of Applied Sciences, Oldenburg, Germany; 2 Department of Obstetrics, Luneburg Hospital, Luneburg, Germany

**Keywords:** latent phase of labor, midwifery care, qualitative research, obstetric services, womancentered care

## Abstract

**INTRODUCTION:**

The latent phase of labor is a critical stage of childbirth, yet access to continuous, needs-based care during this phase remains limited in Germany. Structural challenges in the healthcare system, including a reduced number of obstetric facilities and insufficient financial support for outpatient services, further restrict options for women and midwives.

**METHODS:**

This study aimed to explore the resources available to midwives and the barriers they encounter when providing care and counseling during the latent phase of labor. A qualitative research design was employed. Thirteen semi-structured interviews with midwives were conducted between June 2024 and March 2025. Data were analyzed using qualitative content analysis.

**RESULTS:**

Four main categories emerged: 1) the physiology of the latent phase, 2) decision-making criteria for the location of care, 3) structural conditions in the hospital, and 4) aspects of care improvement. Midwives reported a lack of outpatient options, uncertainties in defining the latent phase, and tensions between guideline recommendations and women’s subjective needs. Structural barriers, such as staff shortages, limited space, and financial disincentives, were highlighted. At the same time, midwives expressed an openness to innovative concepts, including midwife-led outpatient care, designated rooms within clinics, and improved prenatal education.

**CONCLUSIONS:**

Midwives in Germany perceive current support for childbearing women during the latent phase as insufficient. To ensure needs-based care, structural and financial reforms are required, alongside expanded counseling and educational opportunities. Strengthening midwife-led concepts, improving antenatal education, and adapting reimbursement structures, could reduce unnecessary interventions and enhance woman-centered care.

## INTRODUCTION

The latent phase of labor is characterized by irregular uterine contractions and gradual cervical changes and may last several hours or even days^1-3^. Although it represents a normal physiological stage of childbirth, its definition and management vary considerably across clinical guidelines and care settings^3^. While some guidelines define the onset of active labor at 4 cm cervical dilation, others recommend waiting until 5 cm or 6 cm, leading to inconsistencies in admission practices and clinical decision-making^1-4^.

Women often seek hospital admission during the latent phase, due to pain, uncertainty, or anxiety, and the need for reassurance^5-11^. However, early admission has been associated with higher rates of obstetric interventions, including augmentation and cesarean section^11,12^. Consequently, many guidelines recommend delaying hospital admission until active labor is established, provided that maternal and fetal conditions are reassuring^11^. Despite these recommendations, in clinical practice, the management of latent labor remains challenging. Midwives must balance institutional policies, resource constraints, medico-legal considerations, and women’s individual needs. In systems with limited outpatient structures, counseling women to return home during the latent phase could be particularly difficult.

In Germany, maternity care is predominantly hospital-based and has been shaped by increasing centralization and ongoing workforce shortages^13^. In addition to salaried hospital-employed midwives, some maternity units collaborate with self-employed midwives (‘freelance midwives’) who work under independent contractual arrangements and are remunerated on a case-by-case basis through statutory health insurance. These midwives often provide intrapartum care within hospital settings but are not directly employed by the hospital. As a result, their scope of practice and remuneration structures differ from those of salaried staff. In summary, the current financial and organizational frameworks provide only limited support for outpatient care during the latent phase of labour^14,15^. Together, these structural conditions may influence admission decisions and shape midwives’ scope of practice. Although several international studies have examined women’s experiences during the latent phase, less is known about midwives’ perspectives on providing care under these structural constraints, particularly within the German healthcare system. Understanding midwives’ experiences is essential for identifying barriers and potential strategies to improve care during latent labor. Therefore, this study aims to explore midwives’ perspectives on resources and challenges when supporting women during the latent phase of labor within the German maternity care system (Table 1).

**Table 1:** Existing evidence and contribution of the present study on care during the latent phase of labor

*Existing knowledge on this topic*	*Contribution of present study*
The latent phase of labor lacks clear diagnostic criteria and consistent care approaches. In Germany, no structured concept of midwifery care is implemented for this phase, despite professional frameworks including it as an important part of the birth process.	This study explores German midwives’ views on counselling and care during the latent phase. It identifies organizational and structural barriers influencing admission decisions and outlines resources needed to strengthen woman-centered care during the latent phase.

## METHODS

### Design

A qualitative study employing expert interviews was conducted to explore professionals’ perspectives on care during the latent phase of labor. A semi-structured interview guide with open-ended questions enabled adaptive probing to capture emerging themes^16^. All participants provided written informed consent. In accordance with GDPR requirements, interview recordings and anonymized transcripts were stored on researchers’ secure, password-protected laptops.

### Sampling and selection of participants

In Germany, midwives are the primary care providers for physiological births. In 2024, 98% of the 680017 births occurred in a clinical setting. A purposive theoretical sampling strategy was used to capture variation in organizational contexts of maternity care. Participation from midwives working across different levels of perinatal care was intentionally sought, as well as different institutional sizes and urban/rural settings, to explore how structural conditions may shape care during the latent phase of labor. The aim was to interview ten to fifteen midwives who work in an obstetric setting. Midwives who worked in a clinic for at least 50% of their weekly working hours for at least six months were included. To investigate correlations between care capacity for the latent phase and clinic location and size factors, participants were categorized according to their hospital’s perinatal care level. It should be noted that as a result of the 2024 hospital reform, the designation of the care levels changed^17^. During the interviews, the former classification of care levels was used. Participants were recruited via an invitation flyer, which was distributed by email, letter, or in person to maternity wards in the federal states of Lower Saxony, Schleswig-Holstein, Hamburg, and Bremen.

### Data collection

The interview guide was developed by two midwives based on exploratory literature research and refined in a workshop with experienced researchers. Its relevance and clarity were pre-tested by Mother Hood e.V.*,* a parents’ organization advocating for improved obstetric care in Germany. The questions addressed care resources for women in the latent phase, diagnostic and decision-making criteria, counseling content, and professional perspectives on optimal care. Interviews were conducted face-to-face or by telephone by an experienced midwife researcher. Audio recordings were transcribed verbatim^18^, anonymized, and linguistically refined for readability^19^. Transcripts were returned to participants for validation. Quotations used in the manuscript were translated into English, with original quotations and translations presented in Table 2.

**Table 2:** Illustrative quotations from interviews with midwives on care during the latent phase of labor (English translation and original German)

*Interview*	*Quote*	*Original quote*
I8	*‘... I find that difficult because some women are already stressed at 1.5 cm and need support. ... they might not be in labor, but they still need care.’*	*‘Das finde ich schwierig, weil einige sind halt bei einem Zentimeter und noch einem halben Zentimeter Portio schon belastet und brauchen dann auch Begleitung und dann immer zu sagen: “Ja, die sind nicht unter der Geburt! ” Ja, aber sie brauchen jetzt trotzdem Begleitung ...’*
I2	*‘... I think it depends on how well informed they [women] are about it in advance. So, if they somehow know ... and have come to terms with the fact that it can take a long time, then it‘s okay for them. The men tend to be more nervous than the women. But yes, of course, some are impatient or just exhausted and stressed at some point. And maybe that‘s what happens, that they [midwives] just want to help somehow because the women are there [in the hospital], after all.’*	*‘... Ich finde es kommt darauf an, wie gut sie im Vorhinein informiert sind da drüber. Also wenn sie irgendwie wissen ... und damit sich auch abgefunden haben, dass es so lange dauern kann, dass es dann irgendwie für die in Ordnung ist, dass da dann eher die Männer nervöser werden als die Frauen. Aber ja, manche sind natürlich auch ungeduldig oder dann einfach irgendwann erschöpft und belastet und so. Und vielleicht ist da dann auch das, was bei den Hebammen entsteht, dass man irgendwie, weil sie jetzt ja da [im Krankenhaus] sind, dass man dann irgendwie/irgendwie helfen möchte.’*
I8	*‘Yes, legal concerns always play a role ... Even if a woman calls with contractions, you tend to ask her to come in because you have to check ... simply so no one can say that she called and then something was wrong with the baby afterwards ...’*	*‘Ja, da spielen natürlich dann auch immer rechtliche Sachen so eine Rolle. ... Auch wenn eine Frau mit beginnenden Wehen anruft, dass man sie dann eher einbestellt, weil man gucken muss ... Aber dass sie dann doch eher einbestellt wird, einfach damit nicht im Nachhinein gesagt werden kann, sie hat im Kreißsaal angerufen und dann war doch irgendwas mit dem Kind, weil sie nie/ weil man sie nicht einbestellt hat.’*
I5	*‘... and then you can filter out those who are actually doing well ... and those that are just not comfortable at home anymore. Those are the ones who are clearly in need of support, and that support might just mean hearing the baby once, getting an exam and being told that this is still the early stage of labor.’*	*‘ ... und dann kann man die schon mal herausfiltern, die eben/ denen es eigentlich gut geht ... Und ... genau, die, die sagen: Nee, sie sind jetzt zu Hause/ fühlen sich zu Hause nicht mehr wohl. Die nehme ich dann auch deutlich als die wahr, die auch eine Unterstützung brauchen und die Unterstützung kann dann aber auch ausschließlich sein, sie haben einmal das Baby gehört und wir haben/ sind einmal untersucht worden und haben gehört, es ist noch ganz am Anfang oder es ist, was auch immer.’*
I5	*‘... we are a team of freelancers. If we are being honest, we don‘t earn money from the birth itself, but rather from providing care as early as possible, or rather, from providing care for a long time beforehand. And that tends to lead to women being called in relatively early ...’*	*‘... Wir sind ja ein Belegteam. Wenn wir ehrlich sind, verdienen wir nicht an der Geburt, sondern wir verdienen daran, dass wir möglichst/ also, dass wir lange vorbetreuen. Und das führt eben eher dazu, dass man … dann die Frauen vergleichsweise früh einbestellt ...’*
I1	*‘... we also have one or two colleagues who come from other clinics, where the cervix was three or four centimeters dilated for a long time without anything being done. I wasn‘t familiar with that at all. So really, she is saying, “Yeah, she was four centimeters dilated for three hours.” And in principle, that‘s fine. It‘s just the latent phase, that‘s allowed to happen. Of course, you always have to see how the woman is doing, but three years ago, I wouldn‘t have had that. After two hours at the latest, something would have happened, we would have given her the drip [oxytocin] or broken her waters [amniotic sac]. So, there would have already been some sort of intervention to get things moving ...’*	*‘... Wir haben halt auch ein, zwei Kolleginnen, die aus anderen Kliniken kommen, wie lange da halt der Muttermund mal bei drei, vier Zentimetern war, ohne dass was gemacht wurde. Das kannte ich gar nicht. Also wirklich, sie sagt dann: “Joa, die hat drei Stunden lang vier Zentimeter gehabt.” Und ist ja prinzipiell auch in Ordnung. Ist halt Latenzphase, das darf halt sein. Man muss natürlich immer gucken, wie es der Frau damit geht, aber das hätte ich also noch vor drei Jahren gar nicht gehabt. Ich hätte also spätestens nach zwei Stunden nichts passieren, wäre bei uns der Tropf [Oxytocin] mit rangekommen oder die Blase [Fruchtblase] aufgemacht worden oder, oder, oder. Also da wurde schon so interveniert, dass es dann irgendwie auch vorangeht ...’*
I6	*‘It‘s a difficult phase when women can‘t cope ...Because there is nothing worse than 24 hours of painful contractions with no progress ... And that is my secret frustration when I hear the term “latent phase”...’*	*‘Es ist eine schwierige Phase, wenn die Frauen so gar nicht damit umgehen können oder die Wehen so schmerzhaft sind, ohne dass es vorangeht. ... Weil es ist ja nichts schlimmer als 24 Stunden schmerzhafte Wehen und es geht nicht voran. ... Und das ’ist so mein geheimer Frust, wenn ich Latenzphase höre ...’*
I3	*‘So, I think to myself: Let‘s do it the way the WHO recommends, meaning midwife-led facilities for assessing the onset of labor and the latent phase, upstream of the delivery room. ... With appropriate documentation, with appropriate facilities, in case people don‘t want to tackle an extremely long drive, but want to be at the place of birth. That we just make sure that they can use everything that promotes the latent phase.’*	*‘Also ich denke mir so: Lass es uns doch so machen, wie die WHO es empfiehlt: hebammengeleitete Einrichtung zur Einschätzung von Geburtsbeginn und Latenzphase, dem Kreißsaal vorgeschaltet. ... Mit einer angemessenen Dokumentation, ja, mit angemessenen Räumlichkeiten, falls Menschen doch wegen beispielsweise extrem langem Anfahrtsweg, ja, nicht fahren möchten, sondern sich am Ort der Geburt befinden möchten. Dass wir da halt dafür sorgen, dass sie alles, was Latenzphase begünstigt, auch nutzen können, ja.’*
I13	*‘I think it would help many women if their midwives ... could still accompany them at home during the latent phase ... so that this latent phase could somehow also be covered for women outside the hospital. Where they don‘t yet enter this cascade of interventions in the delivery room, in the hospital, and into this expectation, so to speak. And still feel safe and secure. I think some women ... need this sense of security ...’*	*‘Ja, also ich denke, es würde vielen Frauen helfen, wenn ihre Hebammen ... in der Latenzphase auch zuhause begleiten könnten. ... Also, dass diese Latenzphase irgendwie auch für die Frauen draußen/ das gedeckt werden könnte. Wo sie noch nicht so in diese Interventionskaskade im Kreißsaal, im Krankenhaus reinkommen und in diese Erwartungshaltung sozusagen. Und sich trotzdem sicher und aufgehoben fühlen. Ich glaube, das brauchen ja manche Frauen, also viele Frauen, so dieses Sicherheitsbedürfnis ...’*
I8	*‘A completely normal pregnancy doesn‘t actually require a Level 1 facility ... You could say: these women can just stay in a kind of latent phase room and then have their baby there. They don‘t need all the big machinery. I think this would also free up resources for latent-phase care ...’*	*‘Ein ganz physiologischer Schwangerschaftsverlauf muss eigentlich nicht in ein Level-1-Haus. ... Dass man da auch guckt, ja, müssen alle Frauen in den gleichen Kreißsaal oder kann man vielleicht auch innerhalb eines Kreißsaals sagen: okay, diese Frauen dürfen einfach quasi wie in jedem Latenzphasenzimmer bleiben und dann da ihr Kind kriegen. Die brauchen gar nicht die große Maschinerie. Dadurch könnte man glaube ich auch Ressourcen frei machen für die Latenzphasenbetreuung ...’*
I12	*‘Yes, and unfortunately, many of the midwives don‘t speak English either. I would like to see a general training requirement. All colleagues should at least learn the basics, so that they can communicate with all women. Or they should consistently use [translation app for mobile phones] and not just ignore the woman and take it easy on the job.’*	*‘Ja, und viele von den Hebammen sprechen leider auch kein Englisch. Da würde ich mir eine generelle Fortbildungspflicht wünschen. Dass alle Kolleginnen zumindest die Basics lernen, um mit allen Frauen kommunizieren zu können. Oder [App für Übersetzung] verwenden und nicht die Frau einfach abstellen und hier einen gemütlichen Dienst machen. ’*
I6	*‘If doctors are informed about what the latent phase is, what women need during this time and what it‘s all about, then I think that would be fine. I don‘t think we‘ve yet optimized how to support women ... But I wouldn‘t say that there is a one-size-fits-all solution. You always have to weigh the pros and cons, and unfortunately, I don‘t have a magic fix that helps everyone (laughs), and neither do the doctors; they are often unsure themselves ...’*	*‘Eben mit den Ärzten, wenn die informiert sind, was Latenzphase ist und was die Frauen da brauchen und worum es geht, dann wäre das glaube ich auch ganz okay. Wie man sie unterstützt, die Frauen ... ist glaube ich noch nicht optimiert. Aber ich könnte auch nicht sagen, dass es/ es gibt ja auch nicht ein Mittel für alle. Das muss man ja immer abwägen und leider habe ich kein Zaubermittel, was für alle hilft (lacht) und die Ärzte auch nicht, sind da oftmals auch mal unsicher ...’*
I3	*‘... preparing the person accompanying the birth, because they also have immense potential to influence the timing of care, out of uncertainty. That‘s why it‘s so important to look at these resources and also to make sure that this information about the physiology of the latent phase is really part of the preparation, so that this accompanying person is prepared ...’*	*‘... die geburtsbegleitende Person vorzubereiten, weil die ja auch so ein immenses Potenzial hat, den Zeitpunkt der Betreuung mitzubestimmen, aus Unsicherheit. Deswegen ist es total wichtig, diese Ressourcen mit anzuschauen und auch ganz sich zu vergewissern, dass diese Informationen zur Physiologie der Latenzphase auch wirklich Teil der Vorbereitung sind, dass diese Begleitperson vorbereitet ist ...’*

### Analysis

The evaluation employed qualitative content analysis following the approach proposed by Kuckartz et al.^19^, using the MaxQDA 2022^©^ software. As part of the preliminary textual analysis, the anonymized transcripts were reviewed in accordance with the dual control principle, and key topics were identified. These were then summarized and categorized. For further processing, the preliminary results were presented at a research colloquium. A category system was then developed inductively, resulting in four main categories and twelve subcategories. As practicing midwives, the authors were aware of their own professional perspectives and discussed potential biases during analysis.

### Ethical considerations

Ethical approval was granted by the Ethics Committee of Carl von Ossietzky University Oldenburg (file number 2024-049). The study is reported in accordance with the SRQR checklist^20^. All participants received written information about the study and provided informed consent. Participation was voluntary, and confidentiality was ensured throughout the research process.

## RESULTS

In the period between 17 June 2024 and 3 March 2025, thirteen midwives were interviewed in person or via telephone. The interviews lasted between 28 and 65 minutes. Twelve midwives were employed or were freelancers (self-employed) at a hospital in Germany. One midwife specialized in out-of-hospital obstetrics and, following her own concept, provided outpatient care during the latent phase. She was therefore included in the sample to provide an expert perspective on care during the latent phase. Table 3 provides a comprehensive overview of the sample.

**Table 3:** Characteristics of participating midwives and workplace settings, qualitative interview study conducted in Germany, 2024–2025 (N=13)

*No.*	*Care level (hospital)*	*Birth rate/year*	*Spatial capacity*	*Employment*	*Staffing ratio*	*Location*
1	1	2500	5 delivery rooms 4 admission rooms	permanent	4–5 in early shift 3 in late shift 3 in night shift	urban
2	3	1300	4 delivery rooms 1 labor room	permanent	2 in each of 3 shifts	urban
3	Birth center	140	2 delivery rooms 3 additional rooms	freelance	One-to-one support	urban
4	4	240–280	2 delivery rooms 1 labor room	freelance	1 in each of 2 shifts	rural
5	4	280	2 delivery rooms 1 labor room	freelance	1 in each of 2 shifts	rural
6	4	280	2 delivery rooms 1 labor room	freelance	1 in each of 2 shifts	rural
7	2	?	5 delivery rooms	permanent	?	rural
8	1	1850	4 delivery rooms 1 labor room	permanent	3 in each of 3 shifts	not known
9	4	600	4 delivery rooms 1 labor room	permanent	1 in each of 3 shifts 1 (8 a.m. – 4 p.m.)	rural
10	1	2600	6 delivery rooms 3 admission rooms 3 labor rooms	permanent	4 in each of 3 shifts, sometimes additional 1 for cesarean sections	urban
11	4	670	3 delivery rooms 2 admission rooms	freelance	?	rural
12	Not known	700	3 delivery rooms 2 admission rooms	permanent	2 in each of 3 shifts	urban
13	3	600–700	3 delivery rooms 1 admission room	permanent	2 in each of 3 shifts	not known

Four main categories were identified: 1) physiology of the latent phase, 2) decision-making regarding location of care, 3) structural hospital conditions, and 4) care improvement. Table 4 presents the category system with anchor quotes.

**Table 4:** Category system derived from qualitative content analysis, including definitions and anchor quotations, based on interviews with midwives in Germany, 2024–2025 (N=13)

*Main category and definition*	*Subcategory*	*Anchor quote and interview number*
**Physiology of the latent phase** The main code is assigned to statements that refer to the subjective definition of the onset of labor, as well as to circumstances, events, or actions during the latent phase that may have a positive or negative influence on its course.		
	**Definition** This subcode contains statements on the subjective criteria used by healthcare professionals to assess the timing and characteristics of the onset of labor.	***English quote:*** *‘What does the beginning of labor mean to me? ... It’s when women are really in labor. Not just having a contraction every now and then while still being distracted by other things, but when the contractions are regular, and they can really work with them.’* ***Original quote:*** *‘Was ist für mich ein Geburtsbeginn? ... Wenn Frauen richtig in Arbeit gehen. Also das heißt, nicht nur hin und wieder mal eine Wehe haben, aber eigentlich noch abgelenkt sind mit irgendwas Anderem. Sondern in regelmäßigen Wehen, die sie auch annehmen können.’* *(I 5)*
	**Facilitating factors** This subcode is assigned to statements that refer to favorable factors during the latent phase which could have a positive effect on its progression.	***English quote:*** *‘This process – the moment when labor has actually begun – requires such a huge capacity to adapt, to really adjust to the fact that the pregnancy is now truly over and the birth of the baby is about to happen. And that, in my view – and I remember reading this in the literature too – that really needs confirmation first. Like, “What you’re feeling is right!” Exactly. “Yes, these contractions are coming every seventeen minutes, and they’re challenging. Yes, what you’re feeling is exactly right. It feels really uncertain right now, because we don’t know how long this phase will last.” So it’s about meeting the need the women have, the need to share what they’re experiencing and to have it acknowledged.’* ***Original quote:*** *‘Dieser Prozess, dass diese Geburt begonnen hat, … der erfordert ... eine so große Adaptionskraft, Anpassungskraft an: Diese Schwangerschaft ist jetzt wirklich beendet und dieses Ereignis Kindsgeburt steht unmittelbar bevor. Und das braucht aus meiner Sicht und – das erinnere ich auch so aus der Literatur – das braucht erstmal, Tatsache, Bestätigung. “Das, was du empfindest, ist richtig!” Genau. “Ja, diese Kontraktionen kommen mit Abständen von 17 Minuten und die sind herausfordernd. Ja, das ist richtig, was du empfindest, dein Empfinden ist genau richtig. Das fühlt sich total unsicher gerade an, weil wir nicht wissen, wie lange das vielleicht so dauern wird.” Also, dass dieser Bedarf an Mitteilungsbedürfnis, was die Gebärenden haben, einfach befriedigt wird.’* *(I3)*
	**Inhibiting factors** This subcode is used for statements about adverse factors during the latent phase that could negatively affect its progress.	***English quote:*** *‘I often feel that once they’re admitted to the hospital, they come in with this sense of expectation – like, something has to start happening now! And then they’re often not really open to explanations anymore, and that’s when you can easily end up in this cascade of interventions. “I want more pain relief. I want this, and that. I can’t do this anymore. Please, just help me. Do something!’’.’* ***Original quote:*** *‘Ich habe ja oft das Gefühl, wenn sie dann aufgenommen werden, in der Klinik sind, so diesen Erwartungsdruck haben: Da muss jetzt aber auch mal was passieren! Und oft auch sich diesen Erklärungen gar nicht mehr richtig öffnen und man dann oft in so eine Interventionskaskade reinkommt. “Ich möchte noch ein Schmerzmittel. Ich möchte noch das, möchte noch das und ich kann nicht mehr und helfen Sie mir jetzt doch, bitte. Machen Sie irgendwas!”.’ (I13)*
**Decision-making criteria for the location of care** This main code is assigned to statements about the criteria used by healthcare professionals and women in labor to determine where the remaining latent phase should be spent.		
	**Medical history** This subcode is used for statements about medical or social criteria that determine inpatient admission during the latent phase.	***English quote:*** *‘That’s really an issue for me. Especially because these refugee shelters, they’re often 30 or 40 kilometers away. The women come in by ambulance, and the men are brought later somehow, by bus or whatever, often much later. Sending them back home is really difficult. And they don’t have a place there where they actually feel comfortable. ... So they end up being admitted to the ward. I personally don’t see any other option.’* ***Original quote:*** *‘Das ist für mich wirklich so eine Sache. Gerade dadurch, dass diese Flüchtlingsunterkünfte/ sie sind eben oft 30, 40 Kilometer weg. Die Frauen kommen mit dem RTW. Die Männer werden über irgendwen mit irgendwelchen Bussen irgendwann viel später hinterher gebracht. Die wieder nach Hause zu schicken ist eben sehr problematisch. Und die haben da dann auch gar nicht die Unterkünfte, wo sie sich gut fühlen. ... Und dementsprechend sind die dann stationär aufgenommen. Da sehe ich für mich persönlich auch keine Möglichkeit.’* *(I7)*
	Safety and pain perception This subcode comprises statements pertaining to the subjective feeling of safety in a place (be it at home or in a hospital), or specific wishes that the woman in labor has during her latent phase, the fulfilment of which is related to a specific place.	***English quote:*** *‘I’d also say that if she doesn’t feel comfortable at home, she should go to the hospital – no matter what stage she’s in. I think all those relaxing things don’t really help anymore if someone is scared to be at home alone. The tension just gets too strong, and that’s not helpful. So, if being in hospital gives her a real sense of safety, then I think that’s actually more supportive for the birth, even if she ends up being admitted quite early.’* ***Original quote:*** *‘Würde aber auch sagen, dass wenn sie sich zuhause nicht wohl fühlt, dass sie in die Klinik fahren soll, egal in welcher Phase sie ist, weil ich glaube, dass der Nutzen von all den entspannenden Sachen nicht mehr da ist, wenn jemand Angst hat, zuhause ohne Begleitung zu sein. Dann ist die Anspannung so groß, dass es glaube ich nicht förderlich ist und wenn dann jemand ein großes Sicherheitsgefühl daraus schöpft, in der Klinik zu sein, dann ist es geburtsförderlicher glaube ich, auch wenn man dann vielleicht sehr früh aufgenommen wird.’* *(I8)*
**Structural conditions in the hospital** This main code covers all statements relating to the inherent characteristics of inpatient care during the latent phase.		
	**Diagnosis and inpatient admission** This subcode provides detailed descriptions of the diagnostic tools and criteria employed by healthcare professionals to assess the stage of labor. Additionally, it offers comprehensive explanations of the standard procedures for inpatient admission.	***English quote:*** *‘... then the admission check takes place in the delivery room – with a CTG, sometimes an ultrasound, actually almost always an ultrasound, and a vaginal exam, followed by the question: what happens next? If the women are in active labor, they stay in the delivery room. If they’re not, we discuss whether they want to go back home or take a room on the ward.’* ***Original quote:*** *‘… dann findet im Kreißsaal die Aufnahmeuntersuchung statt mit CTG, gegebenenfalls Ultraschall, noch fast immer Ultraschall und vaginaler Untersuchung und der Überlegung, was passiert denn jetzt? Und sind die Frauen aktiv unter Geburt, bleiben sie im Kreißsaal. Sind sie nicht aktiv unter der Geburt, wird besprochen, ob sie eben wieder nach Hause gehen oder aber, ob sie auf der Station ein Zimmer beziehen wollen.’ (I7)*
	**Care capacities and infrastructure** This subcode is assigned when the text refers to the hospital‘s personnel or space capacities.	***English quote:*** *‘... when we had around 3200 births, that was definitely something we had to think about – like, “Okay, how full is the delivery ward right now? Do we need this room? Is someone about to come in who’s already five centimeters dilated?” ... Now, thankfully, since we have a bit fewer births, it’s easier – at least for me personally – to provide better care.’* ***Original quote:*** *‘ … bei 3200 Geburten war das auf jeden Fall noch ein Ding, wo man überlegen musste: “Okay, wie voll ist der Saal jetzt? Brauchen wir den Raum? Kommt noch jemand, der jetzt irgendwie schon fünf Zentimeter ist?” ... Mittlerweile, dafür bin ich sehr dankbar, dass wir ein paar weniger Geburten haben, kann man da ein bisschen/ also ich für mich persönlich kann dann bessere Betreuung bieten.’* *(I1)*
	**Monitoring and birth management** This subcode includes statements regarding interventions performed after inpatient admission during the latent phase due to various indications, as well as statements that allow conclusions to be drawn about the decision-making process regarding these interventions.	***English quote:*** *‘The first thing I’d wish for is time, and that the doctors wouldn’t get impatient. Some don’t, which is wonderful, but others stand there saying, “Why isn’t it progressing? Check again. We need to ...” and I find that really difficult. It puts me in an awkward position, because then I have to explain to the doctor that it’s normal for things not to move forward yet. So, there can be these really tricky interprofessional tensions. It’s exhausting, and it feels like I have to fight for the woman’s space, so that she can move through it at her own pace. And I think that’s really important too – that women have the time they need to enter into the process of giving birth.’* ***Original quote:*** *‘Also das Erste, was ich mir wünschen würde, wäre Zeit, dass die Ärzte nicht unruhig werden. Manche werden das auch nicht, das ist wunderbar und andere, die dann eben da stehen und sagen: “Warum geht es noch nicht voran? Untersuch nochmal. Da muss man jetzt...”, das finde ich richtig schwierig. Da komme ich auch in so eine blöde Stellung, weil ich dann ... dem Arzt erklären muss, dass das normal ist, dass es noch nicht vorangeht. Also da entstehen dann richtig doofe interdisziplinäre ... Verhakungen eben, ... Es ist sauanstrengend und das ist wie, als müsste ich der Frau diesen Raum erkämpfen, dass sie da auch durchgehen darf in ihrem Tempo. Also das finde ich auch wichtig auf der anderen Seite, dass die Frauen ihre Zeit haben, um sich in den Prozess der Geburt zu begeben.’* *(I6)*
**Aspects for care improvement** The code delineates concepts and proposals for enhancing future care for women in labor during the latent phase.		
	**Support and counselling concepts** This subcode includes statements describing possible care and counselling concepts for the latent phase of labor, either in full or in part, which are subjectively assessed as an improvement in care.	***English quote:*** *‘It would be wonderful if every pregnant woman could contact her midwife, who could then come for a home visit to assess whether labor has started or not, and give her some guidance for the latent phase. Ideally, she’d get home visits and support during that phase whenever she needs them. And then, if she’s planning a hospital birth, the midwife could assess whether it’s the right time to go, or if she should stay home a bit longer – so she arrives at the hospital in active labor with a good obstetric assessment, having been perfectly supported at home.’* ***Original quote:*** *‘Also wunderbar wäre, wenn eigentlich jede Schwangere ... ihre Hebamme kontaktieren kann, dass die zu ihr kommt zum Hausbesuch, zur Feststellung: Geburtsbeginn, ja oder nein, und sie ein bisschen anleiten kann für die Latenzphase und dass sie in der Latenzphase Hausbesuche bekommt, wenn sie welche braucht, und Unterstützung. Und dann eben, falls sie die Klinikgeburt plant, dann von der Hebamme die Einschätzung, ist es jetzt der richtige Zeitpunkt zum Losfahren oder sollte sie noch zu Hause bleiben, sodass sie wirklich mit einem guten geburtshilflichen Befund in der aktiven Geburt den Kreißsaal betreten und zu Hause eben perfekt betreut waren.’ (I11)*
	**Restructuring resources** This subcode includes statements describing a restructuring of previous working conditions during the latent phase for midwives, which may lead to future improvements in care.	***English quote:*** *‘I really think there need to be financial incentives for providing care during the latent phase, because if you’re offering serious support, that also means being available at night. ... I actually think an on-call fee makes sense – some colleagues do charge that, and rightly so if they’re truly offering that kind of service. But of course, the women often have to cover those costs themselves, at least partly. ... All I’d need is fair compensation to make it worth staying up all night.’* ***Original quote:*** *‘Also ich finde schon auch, dass man monetäre Anreize [für ambulante Latenzphasen-Betreuung] setzen muss, weil wenn man wirklich eine ernsthafte Latenzphasen-Betreuung anbietet, heißt das ja auch, dass man nachts erreichbar ist. ... Eigentlich finde ich auch eine Rufbereitschaftspauschale, die ja auch manche Kolleginnen durchaus verlangen, zu Recht, wenn sie das dann ernsthaft machen. Und auch auf denen bleiben die Frauen natürlich sitzen, also zumindest teilweise. ... Ich bräuchte nur eine angemessene Entlohnung, um mir die Nächte um die Ohren zu hauen.’* *(I9)*
	**Further training and education of specialist staff** This subcode is assigned to statements describing training measures for professionals involved in supporting pregnant women.	***English Quote:*** *‘First of all – from my perspective, the training needs of all medical staff should be identified. What’s the point if the midwives can support a 56-hour latent phase, but the doctors don’t have the corresponding knowledge? The whole team needs to understand how to handle latent phases, so we can see what we can realistically provide. And we need to be transparent about that. Otherwise, the women can’t focus on anything else if we don’t say, “We just can’t provide that.” I think that’s really important.’* ***Original Quote:*** *‘Erstmal/ Also aus meiner Sicht müsste ... der Fortbildungsbedarf einmal aufgedeckt werden bei ... dem gesamten medizinischen Fachpersonal. Was bringt es uns, wenn die Hebammen eine 56 Stunden lange Latenzphase begleiten können, wenn die ärztlichen Kolleginnen an der Stelle das Wissen nicht dazu haben, ja? Das muss dann das ganze Team auch innehaben, wie sie sich gestalten, die Latenzphasen, um dann halt zu sehen: Was können wir wirklich leisten? Und da auch transparent sein. Sonst können die Frauen sich ja nicht um was anderes kümmern, wenn wir nicht sagen: “Wir können das einfach gar nicht leisten ...” Genau, das finde ich total wichtig.’ (I3)*
	**Prenatal information and education** This subcode is assigned to statements that describe prenatal education measures for pregnant women and their partners.	***English quote:*** *‘... it’s helpful if women just know about the latent phase, because I often observe that I don’t really see an indication for, say, a vaginal exam, but the women push for it anyway. And then, if things haven’t progressed much or only a little, it leads to frustration, and they think, “Ugh, this is so exhausting!” and might end up asking for pain relief sooner. Which is totally fine – every woman should be able to decide for herself. But there’s often this inner expectation like, “It has to move forward now!” ... It’s different when women know that in the beginning, things can just be like that.’* ***Original quote:*** *‘... es ist hilfreich, wenn die Frauen einfach über diese Latenzphase Bescheid wissen, weil ich auch häufig erlebe, dass ich eigentlich gar nicht die Indikation sehe für zum Beispiel eine vaginale Untersuchung, aber die Frauen dann da so drauf drängen, dass man sie dann doch untersucht und das ist dann, wenn es nicht weitergegangen ist oder nur ein bisschen, dass es dann wieder so eine Frustration kommt und sie denken: “Boah, es ist so anstrengend!” und dann vielleicht doch eher zu einem Schmerzmittel gegriffen wird. Was ja total okay ist. Also jede Frau darf selbstbestimmt, finde ich, entscheiden, aber dann doch eher so eine innere Erwartungshaltung da ist von: “Es muss doch jetzt weitergehen!” ... also das ist anders, wenn die Frauen darüber Bescheid wissen, dass es am Anfang halt so sein kann.’* *(I8)*

### The physiology of the latent phase

*Definition*

Midwives described differing criteria for identifying the onset of labor, and emphasized the relevance of women’s subjective perceptions when determining support needs. Many considered the latent phase to be part of the birthing process, rather than a separate preliminary stage:

*‘... I find that difficult because some women are already stressed at 1.5 cm and need support. ... they might not be in labor, but they still need care.’* (I8)

A few midwives expressed uncertainty about defining the latent phase as strictly physiological, particularly when distress or a prolonged duration occurred. A uniform professional definition was perceived as lacking.

*Facilitating factors*

Midwives identified both physical and psychological factors that may promote physiological progression of the latent phase. Commonly mentioned were relaxation measures (e.g. baths, TENS, movement) and continuous emotional support. Encouraging women to view the latent phase as a meaningful part of birth was considered beneficial.

*Inhibiting factors*

Midwives identified subjective and structural factors that may hinder progression, including invasive interventions, exhaustion, misinterpretation of support needs, and language barriers:

*‘... I think it depends on how well informed they [women] are about it in advance. So, if they somehow know ... and have come to terms with the fact that it can take a long time, then it’s okay for them. The men tend to be more nervous than the women. But yes, of course, some are impatient or just exhausted and stressed at some point. And maybe that’s what happens, that they [midwives] just want to help somehow because the women are there [in the hospital], after all.’* (I2)

Overall, midwives described the latent phase as a sensitive transition requiring individualized support, where unmet informational or emotional needs may increase stress.

### Decision-making criteria for the location of care

Once the diagnosis has been made, the woman, her birth companions, and the obstetric staff decide on various criteria regarding where the latent phase should be spent. Differences in assessment emerged regarding the balance between medical criteria, personal preferences, and institutional constraints.

*Medical history*

Midwives assessed parameters such as parity, complications, rupture of membranes, and travel distance when considering admission. Medico-legal concerns were frequently mentioned, and sometimes led to precautionary admissions:

*‘Yes, legal concerns always play a role ... Even if a woman calls with contractions, you tend to ask her to come in because you have to check ... simply so no one can say that she called and then something was wrong with the baby afterward ...’* (I8)

Such defensive practices were described as barriers to needs-based care and were sources of stress.

*Safety and pain perception*

Women’s desire for security and pain relief often influenced admission decisions:

*‘... you can filter out those who are actually doing well ... and those that are just not comfortable at home anymore. Those are the ones who are clearly in need of support, and that support might just mean hearing the baby once, getting an exam, and being told that this is still the early stage of labor.’* (I5)

Midwives noted that the individual sense of safety frequently outweighed clinical criteria. While supporting this need, they acknowledged that limited resources restricted individualized counseling or observation. Overall, the decisions made reflected tensions between guidelines, institutional frameworks, and women’s expectations, requiring midwives to balance professional responsibility and autonomy.

### Structural conditions in the hospital

*Care capacities and infrastructure*

Midwives emphasized that the quality of care largely depended on staff availability and spatial resources. Limited space and understaffing often constrained individualized care. Some midwives mentioned that financial incentives could influence the timing of admission, especially in self-employed settings:

*‘... we are a team of freelancers. If we are being honest, we don’t earn money from the birth itself, but rather from providing care as early as possible, or rather, from providing care for a long time beforehand. And that tends to lead to women being called in relatively early ...’* (I5)

*Monitoring and birth management*

After admission, fetal and maternal parameters were regularly monitored. Participants described a pressure for timely obstetric progress and a variability in tolerance for prolonged latent phases across settings:

*‘... we also have one or two colleagues who come from other clinics, who would tolerate the cervix being three or four centimeters dilated for a long time without anything being done. I wasn’t familiar with that at all. So really, she [colleague] is saying, “Yeah, she was four centimeters dilated for three hours.” And in principle, that’s fine. It’s just the latent phase; that’s allowed to happen. Of course, you always have to see how the woman is doing, but three years ago, I wouldn’t have had that. After two hours at the latest, something would have happened; we would have given her the drip [oxytocin] or broken her waters [amniotic sac]. So, there would have already been some sort of intervention to get things moving ...’* (I1)

Midwives described frustration when prolonged, painful latent phases occurred without progress, while supportive, continuous care was structurally limited:

*‘It’s a difficult phase when women can’t cope ... Because there is nothing worse than 24 hours of painful contractions with no progress ... And that is my secret frustration when I hear the term “latent phase”.’* (I6)

Overall, participants perceive structural and financial constraints as major barriers to individualized, womancentered care. Limited space and staffing, and institutional expectations sometimes lead to early admissions and interventions, even when midwives prefer a more conservative approach.

### Aspects for care improvement

The midwives who participated in the survey set out various ideas and suggestions for improving the quality of care during the latent phase.

*Support and counseling concepts*

Midwives proposed strengthening midwife-led outpatient models, including telephone assessment and home visits, as well as designated hospital rooms for women in the latent phase:

*‘So, I think to myself: Let’s do it the way the WHO recommends, meaning midwife-led facilities for assessing the onset of labor and the latent phase, upstream of the delivery room. ... With appropriate documentation, with appropriate facilities, in case people don’t want to tackle an extremely long drive, but want to be at the place of birth. That we just make sure that they can use everything that promotes the latent phase.’* (I3)

*‘I think it would help many women if their midwives ... could accompany them at home during the latent phase ... so that this latent phase could somehow also be covered for women outside the hospital. Where they don’t yet enter this cascade of interventions in the delivery room, in the hospital, and into this expectation, so to speak, and still feel safe and secure. I think some women ... need this sense of security ...’* (I13)

*Restructuring resources*

Participants recommended improved remuneration for on-call duties and reduced insurance costs for outpatient midwives. They also advocated for more staff and appropriate spaces, noting that low-risk pregnancies do not necessarily require high-level facilities:

*‘A completely normal pregnancy doesn’t actually require a Level 1 facility ... You could say: these women can just stay in a kind of latent phase room and then have their baby there. They don’t need all the big machinery. I think this would also free up resources for latent-phase care ...’* (I8)

*Further training and education of specialist staff*

Participants called for additional professional education about the latent phase and better communication skills, particularly in English, to improve counseling for diverse patient groups:

*‘Yes, and unfortunately, many of the midwives don’t speak English either. I would like to see a general training requirement. All colleagues should at least learn the basics so that they can communicate with all women. Or they should consistently use [translation app for mobile phones] ... and not just ignore the woman and take it easy on the job.’* (I12)

To avoid unnecessary interventions, they also saw a need to raise awareness among doctors about the physiological nature of the latent phase:

*‘If doctors are informed about what the latent phase is, what women need during this time, and what it’s all about, then I think that would be fine. I don’t think we’ve yet optimized how to support women ... But I wouldn’t say that there is a one-size-fits-all solution. You always have to weigh the pros and cons, and unfortunately, I don’t have a magic fix that helps everyone (laughs), and neither do the doctors; they are often unsure themselves ...’* (I6)

*Prenatal information and education*

Midwives emphasized prenatal education as a key factor for coping with the latent phase. To promote realistic expectations and self-efficacy among women and their birth companions, they suggested addressing the topic in antenatal classes, early counseling, and even school-based education:

*‘... preparing the person accompanying the birth, because they also have immense potential to influence the timing of care, out of uncertainty. That’s why it’s so important to look at these resources and also to make sure that this information about the physiology of the latent phase is really part of the preparation, that this accompanying person is prepared ...’* (I3)

Participants also suggested more detailed registration consultations, although time constraints and limited reimbursement often restricted such approaches. Overall, midwives expressed a strong desire for structures that enable continuous, needs-based care in hospital and outpatient settings, highlighting training, prenatal education, and systemic reforms as key strategies.

## DISCUSSION

This study shows that in Germany, access to needs-based care during the latent phase of labor remains limited. Midwives reported frequent early admissions when they perceived increased support needs, despite guidelines recommending inpatient admission only at the onset of active labor. A central concern was the lack of structured outpatient options: women sent home often receive no professional support, as few midwives provide home-based latent-phase care, partly due to financial disincentives and liability concerns^14^. These findings should be interpreted in light of the dual structure of German midwifery employment models, in which salaried hospital-employed midwives work alongside self-employed midwives providing care under independent contractual arrangements. This organizational context may shape both continuity of care and financial incentives during the latent phase.

Midwives described the latent phase as a professional and ethical dilemma. They viewed themselves as advocates for women’s needs, while being constrained by institutional routines and legal frameworks. Diverging definitions of the onset of labor – whether the latent phase is considered part of labor or a preliminary stage – shaped admission practices. These findings align with previous research showing a limited consensus on diagnostic criteria^1-3^ and similar structural challenges in latent labor management in other high-income countries^6,21^.

Participants highlighted supportive measures such as rest, reassurance, and movement as potentially facilitating physiological progress, although effects vary individually. The blurred boundary between supportive and medical interventions, combined with managing exhaustion or distress in a phase defined as physiological, often limited the perceived care options. Similar uncertainties and counseling demands have been reported elsewhere^6,8^. Differences also emerged between clinical assessments and women’s subjective needs, with many women prioritizing safety, which is often associated with hospital monitoring^6,8^. Admission decisions were further influenced by contextual factors, including parity, travel distance, language barriers, and medico-legal concerns. Increasing distances to maternity units following hospital closures may reinforce precautionary admissions, as travel time becomes an additional safety consideration.

Structural and financial conditions were described as major determinants of care quality. Adequate staffing and space were considered prerequisites for personalized support, while high workloads and limited infrastructure restricted counseling and individualized management. Financial incentives shaped practice in different ways: in some settings, early care provision was economically advantageous, whereas in others, remuneration decreased when caring for several women simultaneously^22,23^. Such inconsistencies may inadvertently promote early admission and intervention cascades. Although participants were recruited from facilities with different levels of perinatal care, no clear pattern of attitudes towards latent-phase management emerged according to care level alone. Rather, midwives across settings consistently described staffing levels, workload, and the number of women requiring simultaneous care as key factors shaping admission decisions and the ability to provide individualized support. Given the small and heterogeneous sample, these observations should be interpreted cautiously.

Participants proposed strengthening midwife-led outpatient models, including telephone assessments, home visits, or designated latent-phase rooms within hospitals. International examples, such as Ireland’s Labor Hopscotch Framework^24^, indicate that structured, low-intervention environments may improve resource allocation and women’s experiences. However, sustainable financing and clear liability coverage were regarded as essential prerequisites for implementation in Germany. Antenatal education was identified as another key strategy. Early, realistic information about the latent phase – provided in classes, consultations, or registration interviews – may reduce anxiety and unnecessary early hospital attendance. Yet such counseling is frequently underfunded and inconsistently reimbursed^25^.

Midwives also called for professional training to strengthen the understanding of the latent phase among both midwives and doctors. Improved knowledge of its physiology could reduce pressure for interventions and support shared decision-making. Language skills were additionally regarded as essential for equitable care in increasingly diverse patient populations.

Overall, the findings illustrate how structural, professional, and emotional factors intersect in shaping care during the latent phase. Systemic reforms – ensuring adequate staffing, appropriate facilities, fair remuneration, and clear liability frameworks – appear to be central to improving woman-centered care. Integrating midwife-led continuity concepts and strengthening antenatal education should enhance autonomy and satisfaction while reducing unnecessary interventions. Future research should evaluate the implementation and outcomes of innovative latent-phase care concepts, ideally through co-design approaches involving women, midwives, and policy makers^26^.

Although this study is situated in the German healthcare system, similar challenges regarding latent labor management, use of resources, and midwife-led concepts have been described across international settings^7-11^. The findings, therefore, contribute to ongoing discussions about strengthening midwife autonomy and the continuity of care within maternity systems.

### Strengths and limitations

This study is the first to explore midwives’ perspectives on caring for women during the latent phase of labor in Germany. The main strength of this study lies in its exploration of a largely unexamined area of maternity care. The inductively developed category system can inform further research and guide policy discussions on how to strengthen midwife-led, woman-centered support during the latent phase. While it provides valuable insights into professional experiences and structural barriers, several limitations must be acknowledged. As the study focused exclusively on midwives working within the German healthcare system and was conducted in German, transferability to other countries is limited due to differing legal, financial, and organizational frameworks for midwifery care.

The qualitative design involved a small, non-representative sample of thirteen midwives. The findings, therefore, reflect subjective viewpoints and cannot be generalized to all midwives or clinical settings. The sample size also did not allow meaningful comparisons between participants working in different levels of perinatal care. However, the aim of qualitative research is to gain an in-depth understanding, rather than statistical representativeness^27^, and the sample was purposefully diverse in terms of professional background and institutional context. Nevertheless, the uneven distribution of participants across different perinatal care levels limits the ability to compare hospital types, with midwives from high-risk facilities being underrepresented. Despite these constraints, participants provided detailed accounts of their working environments and identified potential links between structural conditions and care quality.

### Implications

**Figure 1:**
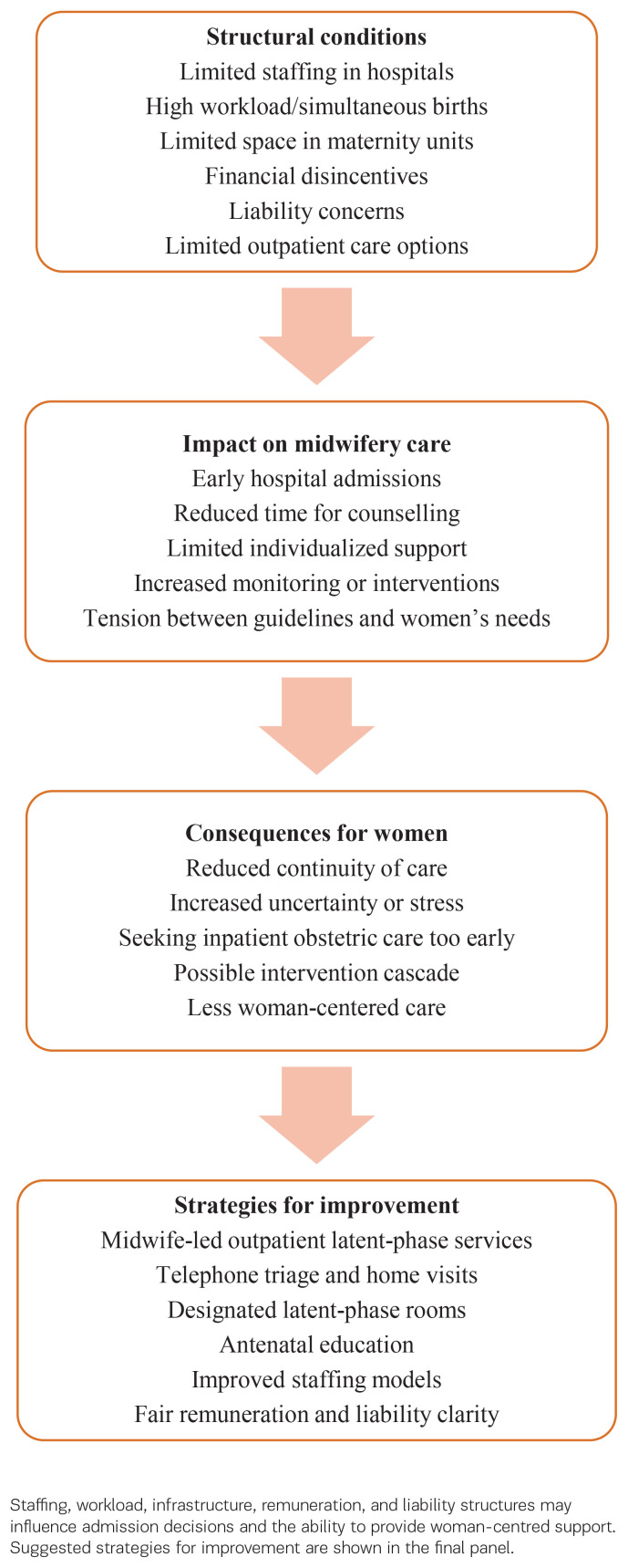
Conceptual summary of how structural conditions shape counselling and care during the latent phase of labour in Germany.

Midwives should be enabled and supported in providing guidance and continuity of care during the latent phase of labor across community and hospital settings. Strengthening midwife-led outpatient care, antenatal education, and designated latent-phase spaces within maternity units could reduce unnecessary interventions, improve access to appropriate care during the latent phase, and promote woman-centered care. Sustainable remuneration models and clear liability frameworks are essential to make such approaches feasible in routine practice. Practical priorities include piloting midwife-led outpatient latent-phase services, integrating preparation for the latent phase into antenatal education, ensuring staffing models that allow sufficient time for counseling and individualized support, expanding equitable access to latent-phase care, and improving access to translation resources for women with limited proficiency. Further research should examine the feasibility, acceptability, and outcomes of these approaches, while policymakers should adapt reimbursement structures to better support continuity of care and clearly define midwifery roles during the early stage of labor. The structural factors influencing care during the latent phase of labor and potential strategies for improvement are illustrated in Figure 1.

## CONCLUSIONS

This qualitative study identified key resources and barriers shaping midwives’ counseling and care during the latent phase of labor in Germany. The findings indicate that structural and financial conditions strongly influence both admission decisions and the quality of woman-centered support. Staffing levels, workload, and spatial limitations were described as major factors determining whether midwives could provide individualized care. Self-employed midwives working in hospitals also face financial disincentives that may restrict their ability to offer comprehensive latent-phase support.

These challenges highlight the need for structural reforms to ensure fair remuneration, clear liability frameworks, and sustainable staffing models. Midwives expressed strong interest in innovative, midwife-led approaches – such as outpatient counseling, home visits, and designated latent-phase rooms within hospitals – that could align women’s needs for safety and continuity with professional and institutional realities. Enhanced prenatal education and well-resourced registration consultations may also help reduce unnecessary early admissions.

Future initiatives should evaluate the feasibility and outcomes of co-designed care models within the German context. Strengthening midwife-led continuity of care and expanding outpatient counseling options could improve women’s experiences during the latent phase, reduce intervention rates, and contribute to a more balanced and sustainable maternity care system. Additional training for obstetric staff on managing the latent phase could further enhance the quality of care.

Reorganizing latent-phase care is not merely a clinical issue, but a structural and policy challenge. Supporting midwives in providing continuous, needs-based care during latent labor will represent a key step towards more sustainable and woman-centered maternity services in Europe.

## Data Availability

Due to privacy and data protection restrictions, the datasets generated and analyzed during the current study are not publicly available, but are available from the corresponding author on reasonable request.
